# Influence of Klotho gene polymorphisms on vascular gene expression and its relationship to cardiovascular disease

**DOI:** 10.1111/jcmm.12710

**Published:** 2015-11-05

**Authors:** Javier Donate‐Correa, Ernesto Martín‐Núñez, Rafael Martínez‐Sanz, Mercedes Muros‐de‐Fuentes, Carmen Mora‐Fernández, Nayra Pérez‐Delgado, Juan F. Navarro‐González

**Affiliations:** ^1^ Research UnitUniversity Hospital Nuestra Señora de CandelariaSanta Cruz de TenerifeSpain; ^2^ Cardiovascular Surgery ServiceHospital Universitario de CanariasSanta Cruz de TenerifeSpain; ^3^ Clinical Biochemistry ServiceUniversity Hospital Nuestra Señora de CandelariaSanta Cruz de TenerifeSpain; ^4^ Nephrology ServiceUniversity Hospital Nuestra Señora de CandelariaSanta Cruz de TenerifeSpain

**Keywords:** Klotho, vascular expression, single nucleotide polymorphisms

## Abstract

Klotho protein has been associated with beneficial effects that contribute to the maintenance of cardiovascular health. Diverse studies suggest that alterations in the levels of this molecule may be associated with pathophysiological abnormalities that result in increased cardiovascular risk. The primary aim of this proof‐of‐concept study was to analyse the existence of a potential link between *Klotho* gene polymorphisms and the expression level of this gene in the vascular wall, and additionally with the incidence of cardiovascular disease and cardiovascular risk factors. Our results indicate that the variant G‐395A, located in the promoter region, influences *Klotho* gene vascular expression and is associated with the incidence of diabetes. Similarly, the exonic variant KL‐VS was associated with the incidence of atherosclerotic vascular disease and coronary artery disease. Moreover, vascular expression levels of *Klotho* were related with the incidence of diabetes mellitus and coronary artery disease. These findings, which need to be confirmed in larger studies, suggest a potential role of Klotho in the pathogenesis of vascular damage.

## Introduction

Klotho protein was first related to cardiovascular disease in the experiments by Kuro‐o *et al*.^1^, where a Klotho deficient‐mice model showed accelerated arteriosclerosis associated with extensive medial calcification of the aorta, as well as both medial calcification and intimal thickening of medium‐sized muscular arteries. In addition, these animals exhibited impaired angiogenesis and vasculogenesis 2, 3 and endothelial dysfunction 4, 5, which could be ameliorated by *in vivo* gene delivery of the *Klotho* gene or by parabiosis with a Klotho wild‐type specimen 6. Other experimental models confirmed the existence of Klotho protective effects upon vascular system that include the maintaining of endothelial wall homeostasis and the promotion of vascular health 4, 6, 7 whereas Klotho deficiency triggers endothelial dysfunction and vascular calcification 7, 8.

Klotho is a type 1 transmembrane protein expressed predominantly in the kidneys where acts as a co‐receptor for the binding of the phosphatonin fibroblast growth factor‐23, playing a central role regulating phosphate excretion and calcitriol synthesis. Most of the transmembrane form of the protein is localized extracellularly, and contains two homologous domains (KL1 and KL2). The transmembrane form can be shed from the cell surface, and the shed form can be detected in serum and urine. Importantly, recent studies have confirmed the expression of the *Klotho* gene and protein in human vascular tissue 9, 10 suggesting that vascular Klotho may play a significant role in maintaining cardiovascular health, with reduced Klotho being associated with cardiovascular disease 10, 11.

*Klotho* gene, located in the largest arm of human chromosome 13, contains five exons and four introns that span approximately 50 kb. Genetic variation studies in humans have determined that *Klotho* gene polymorphisms might be associated with longevity and cardiovascular disease 12, 13, 14, 15, 16 However, none of those studies have considered the vascular expression of *Klotho* as a possible determinant of the association of these single nucleotide polymorphisms (SNPs) with cardiovascular disease. In this work, we have tested the influence of *Klotho* gene variants on *Klotho* gene expression levels in human vascular tissue and their association with cardiovascular disease and cardiovascular risk factors.

## Materials and methods

### Patients

One hundred and five consecutive patients who underwent elective cardiac surgery were included in this study (coronary artery bypass surgery in 79 patients and replacement valvular surgery in 26 patients). Informed consent was obtained from all patients, and thoracic aorta specimens were obtained in all cases. The study was approved by the Ethics Committee of Hospital Universitario Nuestra Señora de Candelaria and Helsinki principles were followed.

### Clinical characteristics

In this study, cardiovascular disease and cardiovascular risk factors were considered for analysis. Cardiovascular disease included aortic stenosis and cardiac valve calcification (both determined by echocardiography); coronary artery disease (determined by coronary angiography) and atherosclerotic vascular disease (defined as the presence of coronary artery disease and/or carotid atheromatosis and/or peripheral vascular disease). Cardiovascular risk factors included LV hypertrophy (determined by echocardiography); diabetes mellitus (defined according to the criteria from the American Diabetes Association); dyslipidaemia (defined as elevated serum cholesterol and/or triglycerides); and arterial hypertension (defined as systolic blood pressure higher than 140 mmHg and/or diastolic blood pressure higher than 90 mmHg). In order to avoid potential confounding factors, no patient had renal insufficiency, defined as an estimated glomerular filtration rate lower than 60 ml/min/1.73 m^2^.

### SNP genotyping

Genomic DNA was purified from peripheral blood cells using a commercial kit (QIAamp DNA Blood Mini Kit; Qiagen, Hilden, Germany). In this study, we analysed two SNPs in *Klotho* loci: rs9536314 (F352V) and rs1207568 (G‐395A). Genotyping was performed using TaqMan genotyping reaction (Applied BioSystems, Foster City, CA, USA). DNA was amplified on a Veriti^™^ Thermal Cycler (Applied BioSystems; 95°C for 10 min., followed by 40 cycles of 95°C for 15 sec., and 60°C for 1 min.), and fluorescence was detected in a 7500 Fast Real‐Time PCR System (Applied BioSystems). TaqMan assays employed were C‐2983037‐20 for rs9536314 and C‐7604792‐10 for rs1207568 (Applied BioSystems). To assess genotyping reproducibility a random 5% selection of the sample was regenotyped with a 100% of genotypic concordance.

### RNA extraction and gene expression analysis

Aortic samples (about 10 mg in average) were recovered during surgery and immediately placed in RNAlater^®^ (Ambion (Europe) Limited, Huntingdon, Cambridgeshire, UK) solution and stored at 4°C for subsequent RNA extraction. Total RNA was isolated from tissues kept on ice after complete homogenization in TRI Reagent^®^ (Sigma‐Aldrich, St. Louis, MO, USA) employing TissueRuptor (Qiagen) and further purified using RNeasy Mini kit (Qiagen), according to manufacturer's specifications. Quality of extracted RNA was tested using an Experion^™^ Automated Electrophoresis System (Bio‐Rad Laboratories, Hercules, CA, USA) and quantified using a Thermo Scientific NanoDrop 2000 spectrophotometer (Thermo Scientific Nanodrop, Wilmington, DE, USA). cDNA was obtained using a High Capacity RNA‐to‐cDNA kit (Applied BioSystems) according to manufacturer's instructions to be used in quantitative real‐time PCR (qRT‐PCR). Transcripts encoding for *Klotho* and *glyceraldehyde 3‐phosphate dehydrogenase* (*GAPDH*) were measured by TaqMan qRT‐PCR with TaqMan Fast Universal PCR Master Mix (Applied BioSystems). TaqMan Gene Expression Assays for each transcript (Hs00183100_m1 [*Klotho*], and Hs99999905_m1 [*GAPDH*]) were analysed in a 7500 Fast Real‐Time PCR System (Applied BioSystems). The level of target mRNA was estimated by relative quantification using the comparative method 2^−ΔΔCt^ by normalizing to *GAPDH* expression. Quantification of each sample was tested in triplicate, and a corresponding non‐reverse transcriptase reaction was included as a control for DNA contamination.

### Statistical analysis

Statistical analysis was performed using GraphPad InStat (GraphPad Software, San Diego, CA, USA). The fold‐change of target genes expression was calculated with Data Assist^™^ v2.0 Software (Applied BioSystems). Gene expression values were logarithmically transformed. A chi‐squared test was performed to determine whether genotype frequencies were in Hardy–Weinberg equilibrium. For the gene expression continuous variable, we performed an unpaired *t*‐test. The significance of differences for the categorical variables was analysed by using the Fisher's exact test. A *P* < 0.05 was considered statistically significant.

## Results

One‐hundred and five subjects (31 females, 74 males) aged 67 ± 11 years (ranged from 46 to 83 years) were included in the present study. The incidence of cardiovascular disease was: aortic stenosis, 44.7%; valvular calcification, 25.7%; atherosclerotic vascular disease, 80.9%; coronary artery disease, 70.5%. Regarding cardiovascular risk factor, the incidence of LV hypertrophy, diabetes mellitus, hypertension and dyslipidaemia was 28.6%, 43.8%, 60.9% and 62.9%, respectively.

**Figure 1:**
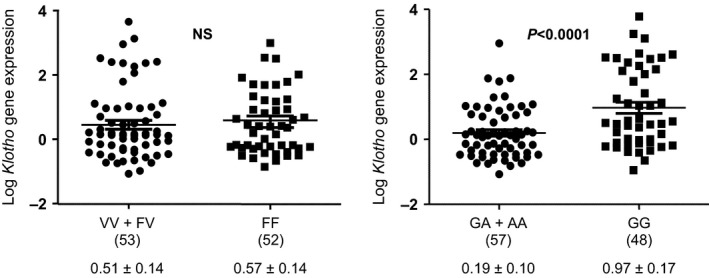
Relative *Klotho* mRNA expression levels from aortic samples segregated by genotype and comparing individuals homozygous for the protective allele to individuals with all other genotypes. (N)= number of subjects. NS: not significative. Gene expression data are expressed as mean ± SD.

Genotype distributions of Klotho KL‐VS FF, FV and VV were 0.495 (*N* = 52), 0.39 (*N* = 41) and 0.114 (*N* = 12), respectively, and satisfied the Hardy–Weinberg equilibrium (chi‐squared test *P* > 0.05). Patients who carried the minor allele showed a significant greater incidence of atherosclerotic vascular disease (90.5%) compared to subjects with FF homozygotics (69.2%) (*P* < 0.01; Table 1). In addition, the difference between these groups regarding the incidence of coronary artery disease almost reached statistical significance (*P* = 0.08). Coronary artery disease was present in 41 of the 53 subjects allele V carriers (77.4%), whereas the entire group of 12 patients (100%) with the VV variant presented coronary disease. There were no differences in gene expression levels for the exonic KL‐VS variant carriers (Fig. 1).

**Table 1:** Comparison of clinical characteristics according to G‐395A and F352V genotypes. Fisher's exact test

	F352V (rs9536314)			G‐395A (rs1207568)		
	VV + FV (53)	FF (52)	*P*	GA + AA (64)	GG (41)	*P*
AS	25 (47.2)	22 (42.3)	NS	27 (42.2)	19 (46.3)	NS
VC	16 (30.2)	11 (21.2)	NS	16 (25)	11 (26.8)	NS
DM	26 (49.1)	20 (38.5)	NS	34 (53.1)	13 (31.7)	<0.05
DP	30 (56.6)	34 (65.4)	NS	41 (64.1)	24 (58.5)	NS
HT	38 (71.7)	28 (53.8)	NS	37 (57.8)	28 (68.3)	NS
AT	48 (90.5)	36 (69.2)	<0.01	53 (82.8)	32 (78)	NS
CAD	41 (77.4)^a^	32 (61.5)	0.08	47 (73.4)	27 (65.9)	NS
LVH	16 (30.2)	14 (26.9)	NS	16 (25)	14 (34.1)	NS

a All 12 subjects with VV genotype presented CAD.

AS: aortic stenosis; VC: valvular calcification; DM: diabetes mellitus; DP: dyslipidaemia; HT: hypertension; AT: atherosclerosis; CAD: coronary artery disease; LVH: left ventricular hypertrophy; NS: not significative.

The G‐395A variant, which is located in the promoter region, presented the following genotype distribution: GG, 0.457 (*N* = 48); GA, 0.39 (*N* = 41); and AA, 0.15 (*N* = 16), and satisfied the Hardy‐Weinberg equilibrium (chi‐squared test *P* > 0.05). Our results show that this polymorphism did not share association with any of the cardiovascular features. However, regarding cardiovascular risk factors, the incidence of diabetes mellitus was significantly higher in allele A carriers (53.1%) compared to the subjects homozygous for G (31.7%), *P* = 0.001 (Table 1). Expression levels of *Klotho* in vascular tissue was reduced in allele A carriers compared to GG subjects [Log arbitrary units (a.u.) = 0.1959 ± 0.1001 *versus* 0.9713 ± 0.1725; *P* < 0.0001] (Fig. 1).

**Figure 2:**
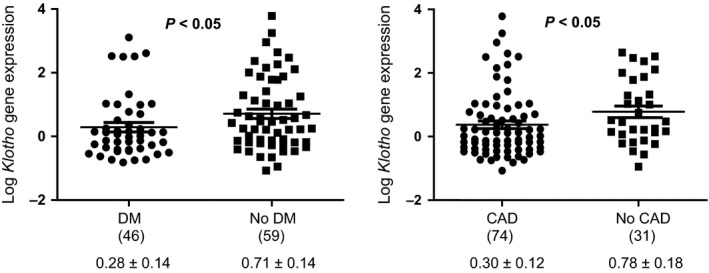
Comparison of clinical characteristics according to relative Klotho gene expression levels in aortic samples. (N) = number of subjects. DM: diabetes mellitus; CAD: coronary artery disease; AT: atherosclerosis; NS: not significant. Gene expression data are expressed as mean ± SD. Values are analysed by *t*‐test.

When analysing vascular *Klotho* expression levels according to the presence of cardiovascular disease and cardiovascular risk factors, we observed that individuals with coronary artery disease had a significantly lower expression of *Klotho*: Log a.u. in coronary artery disease patients, 0.30 ± 0.12 *versus* non‐coronary artery disease patients, 0.78 ± 0.18 (*P <* 0.05; Table 2). Patients with atherosclerotic vascular disease showed a non‐significant trend towards lower vascular *Klotho* mRNA levels as compared with subjects without atherosclerosis: Log a.u. in atherosclerotic patients, 0.41 ± 0.11 *versus* non‐atherosclerotic patients, 0.82 ± 0.24 (*P* = 0.11). Regarding cardiovascular risk factors, vascular *Klotho* expression levels were significantly lower in diabetics as compared with non‐diabetic subjects: Log a.u. in diabetics, 0.28 ± 0.14 *versus* 0.71 ± 0.14 in non‐diabetics, *P <* 0.05 (Fig. 2).

**Table 2:** Comparison of relative *Klotho* gene expression levels in vascular samples according to the presence or absence of different clinical characteristics

	Presence, mean ± SEM (*N*)	Absence, mean ± SEM (*N*)
AS	0.5456 ± 0.1053 (39)	0.4627 ± 0.1055 (66)
VC	0.5394 ± 0.1058 (79)	0.4909 ± 1.0554 (26)
DM	0.2897 ± 0.1458 (46)	0.7115 ± 0.1474 (59)
DP	0.5092 ± 0.1089 (64)	0.4851 ± 0.1141 (41)
HT	0.3684 ± 0.0984 (66)	0.4651 ± 0.1131 (39)
AT	0.2165 ± 0.0942 (59)	0.2645 ± 0.1064 (46)
CAD	0.3028 ± 0.1218 (74)	0.7818 ± 0.183 (31)
LVH	0.5621 ± 0.0984 (31)	0.5842 ± 0.1036 (74)

AS: aortic stenosis; VC: valvular calcification; DM: diabetes mellitus; DP: dyslipidaemia; HT: hypertension; AT: atherosclerosis; CAD: coronary artery disease; LVH: left ventricular hypertrophy; NS: not significative.

## Discussion

In the present proof‐of‐concept study we aimed to evaluate the genetic contribution of the *Klotho* KL‐VS and G‐395A variants to the incidence of cardiovascular disease, its relationship with cardiovascular risk factors, and the influence in the expression level of this gene in human aortic samples.

We have analysed two SNPs located in the codifying (KL‐VS) and in the promoter (G‐385A) regions of the *Klotho* gene, which could modify the functionality and/or the expression of this protein, respectively. The functional KL‐VS variant is composed of six SNPs in perfect linkage disequilibrium, two of which result in amino acid substitutions F352V and C370S altering *in vitro* secretion and activity of the protein and thus, its functionality 12, 17. The single variant F352V, which is used to tag the KL‐VS haplotype, implies the substitution of phenylalanine to valine at position 352. The functional variant KL‐VS is prevalent in the general population (15.7%) and has been linked with increased cardiovascular risk, promoting elevated systolic blood pressure, serum cholesterol and cardiovascular disease in Caucasians and African Americans 12, 13, 16, 17, 18. The single variant G‐395A is located in the promoter region, and has been associated with reduced cardiovascular rates in Korean women 16.

In this work, we have observed that the functional variant KL‐VS is related to differences in the incidence of atherosclerotic vascular disease. Thus, the incidence of this disease in subjects with the V allele was significantly higher than the incidence observed in patients with the FF variant. In addition, we also observed a trend towards higher incidence of coronary artery disease in subjects with the V allele. Previous studies in humans have reported that this variant is associated with HDL‐cholesterol, systolic blood pressure and other cardiovascular risk factors 12, 13, 17, 19, 20. The findings of our study confirm the previous suggestion that the minor allele of the KL‐VS variant may be related to cardiovascular risk. Otherwise, although Klotho is involved in the homeostasis of calcium and phosphorus, we have not found any association between this polymorphism with vascular calcification, in agreement with the previously reported candidate gene study from the Framingham Offspring Cohort 21.

Interestingly, we have found that *Klotho* gene polymorphism G‐395A, located in the promoter region, is related to *Klotho* expression levels in human vascular tissue. Although previous works have speculated that the presence of allele A in this polymorphism reduces the binding of transcription factors and hence expression levels 22, our findings show for the first time that human vascular expression levels of Klotho are reduced in subjects with allele A as compared to GG subjects. These differences in the expression levels were not observed for the KL‐VS variant.

An association of Klotho variants with coronary artery disease has been reported previously 13, 19. However, the relationship between soluble Klotho concentrations and vascular Klotho expression levels with human coronary artery disease was not elucidated until recently 11. This work concluded that patients with significant coronary heart disease presented lower soluble concentrations of Klotho, as well as reduced levels of Klotho expression in thoracic aorta. Moreover, this association was independent of traditional cardiovascular risk factors 11. Unfortunately, *Klotho* gene polymorphisms were not analysed in that previous study. The findings of the present work suggest that variability of *Klotho* genotype may be related to vascular Klotho expression and coronary artery disease. Low Klotho levels has been suggested as a key factor in the development of endothelial dysfunction 23, which is involved in the initiation, progression and complications of athreosclerosis 24. In the present work, vascular *Klotho* mRNA levels were significantly lower in subjects with coronary artery disease, and interestingly, as reported above, the presence of allele A variant of the G‐395A *Klotho* gene polymorphism was associated with lower vascular Klotho expression. Experimental models have confirmed protective effects of Klotho upon vascular system. Several mechanisms have been proposed to explain these beneficial effects, including stimulation of calcitriol and nitric oxide synthesis, and suppression of Wnt signalling and oxidative stress 4, 25. Thus, it is possible to speculate that the allele A variant of the G‐395A *Klotho* gene is associated with lower vascular *Klotho* expression, which would promote the development of endothelial dysfunction.

Finally, another interesting observation was the significantly higher incidence of diabetes mellitus in subjects with the allele A variant, a difference that could be related to the lower levels of Klotho expression. Depleted protein expression of Klotho has been described in pancreatic islets in animal models of diabetes, and moreover, *in vivo* expression of Klotho in pancreatic β cell preserved its functionality and attenuated the development of diabetes in db/db mice 26. In addition, clinical studies have shown that serum levels of Klotho are significantly decreased in patients with diabetes compared with non‐diabetic subjects 27. Although it is necessary to interpret these data with caution, these findings suggest that Klotho may be involved in the pathogenesis of diabetes mellitus.

Although our study provides interesting information about Klotho system, we recognized several limitations. First, and the major limitation of this study, is the lack of a true control group. However, this work was designed as a proof‐of‐concept study, and therefore, a small number of participants were included to test our main assumption about the potential influence of *Klotho* gene polymorphisms on *Klotho* gene expression levels in human vascular tissue. The results of the study indicate that this assumption can be valid for generating new hypothesis. Second, the reduced vascular Klotho expression does not mean that these patients had Klotho insufficiency. In fact, since our patients had no renal insufficiency, and the kidney is the principal contributor of circulating Klotho 28, we assume that circulating Klotho levels are normal. However, the presence of normal (or not reduced) circulating Klotho does not mean that the expression of Klotho in other tissues (including the vascular wall) is normal. Thus, it is possible that in spite of the presence of normal circulating Klotho concentrations, the vascular expression of Klotho may be reduced due to different factors, including Kotho genetic variants. Unfortunately, due to logistical problems, a reliable serum sample could not be obtained for measuring circulating Klotho concentrations. Third, due to the lack of a reliable serum sample, other factors with potential interest and potential relationship with vascular Klotho expression and Klotho polymorphisms could not be analysed, inlcuding fibroblast growth factor 23 and serum vitamin D levels. Fourth, since the vascular wall samples were very small (about 10 mg average weight), we were not able to perform additional analysis, such us the Klotho expression levels in different cell types (endothelial or smooth muscle cells) or the measurement of Klotho protein expression and the potential relationship with Klotho mRNA changes.

In conclusion, to the best of our knowledge, this is the first study which links *Klotho* gene polymorphisms with the vascular expression of this gene. Only one previous study determined associations of *Klotho* intronic SNPs with increased expression levels, but those data were obtained from Epstein‐Barr virus‐transformed lymphoblastoid cell lines derived from HapMap samples as a part of the Genevar Project and the Wellcome Trust Sanger Institute 29. In that work, Bostrom *et al*. hypothesize that this increase linked to several haplotypes may be responsible of a protective role in the progression of renal disease in African‐Americans. Our results indicate that the polymorphic variability in *Klotho* gene may be a factor influencing tissue expression levels of this protein as well as in the incidence of diabetes mellitus, atherosclerotic vascular disease and coronary heart disease.

## Conflicts of interest

The authors declare no conflict of interest.
